# Mobile Phone Access and Implications for Digital Health Interventions Among Adolescents and Young Adults in Zimbabwe: Cross-Sectional Survey

**DOI:** 10.2196/21244

**Published:** 2021-01-13

**Authors:** Aoife M Doyle, Tsitsi Bandason, Ethel Dauya, Grace McHugh, Chris Grundy, Stefanie Dringus, Chido Dziva Chikwari, Rashida A Ferrand

**Affiliations:** 1 MRC International Statistics and Epidemiology Group London School of Hygiene & Tropical Medicine London United Kingdom; 2 Biomedial Resarch and Training Institute Harare Zimbabwe; 3 Department of Clinical Research London School of Hygiene & Tropical Medicine London United Kingdom

**Keywords:** adolescent, young adult, young person, young people, cross-sectional studies, humans, female, male, mobile phone, smartphone, cell phones, technology, internet, safety, health-related internet use, Zimbabwe

## Abstract

**Background:**

Mobile phones may help young people (YP) access health information and support health service engagement. However, in low-income settings there is limited knowledge on YP’s phone and internet access to inform the feasibility of implementing digital health interventions.

**Objective:**

We investigated access to information and communication technologies among adolescents and young adults in Zimbabwe.

**Methods:**

A cross-sectional population-based survey was conducted from October to December 2018 among YP aged 13-24 years in 5 communities in urban and peri-urban Harare and Mashonaland East, Zimbabwe. Consenting YP completed a self-completed tablet-based questionnaire on mobile phone ownership and use, and use of the internet. The primary outcome was the proportion who reported owning a mobile phone. Secondary outcomes included phone and internet access and use behavior, and ownership and use of other technological devices. Multivariable logistic regression was used to investigate factors associated with mobile phone ownership and with internet access, with adjustment for the one-stage cluster sampling design. A priori exploratory variables were age, sex, marital status, and urban/peri-urban residence.

**Results:**

A total of 634/719 (88.2%) eligible YP, mean age 18.0 years (SD 3.3) and 62.6% (397/634) females, participated. Of the YP interviewed, 62.6% (396/633; 95% CI 58.5-66.5) reported owning a phone and a further 4.3% (27/633) reported having access to a shared phone. Phone ownership increased with age: 27.0% (43/159) of 13-15-year olds, 61.0% (72/118) of 16-17-year olds, 71.5% (103/144) of 18-19-year olds, and 84.7% (171/202) of 20-24-year olds (odds ratio [OR] 1.4, 95% CI 1.3-1.5) per year increase. Ownership was similar among females and males: 61.0% (236/387; 95% CI 55.6-66.1) versus 64.8% (153/236; 95% CI 57.8-71.2), age-adjusted OR 0.7 (95% CI 0.5-1.1); higher in those with secondary level education compared to primary or no education: 67.1% (346/516; 95% CI 62.6-71.2) versus 26% (21/82; 95% CI 16.4-37.7), age-adjusted OR 2.3 (95% CI 1.1-4.8); and similar across other sociodemographic factors. YP reported that 85.3% (361/423) of phones, either owned or shared, were smartphones. Among phone owners, the most commonly used phone app was WhatsApp (71.2%, 282/396), and 16.4% (65/396) reported having ever used their phone to track their health. A total of 407/631 (64.5%; 95% CI 60.3-68.5) currently had access to the internet (used in last 3 months on any device) with access increasing with age (OR 1.2, 95% CI 1.2-1.3 per year increase). In age-adjusted analysis, internet access was higher among males, the unmarried, those with a higher level of education, phone owners, and those who had lived in the community for more than 1 year. The aspect of the internet that YP most disliked was unwanted sexual (29.2%, 136/465) and violent (13.1%, 61/465) content.

**Conclusions:**

Mobile phone–based interventions may be feasible in this population; however, such interventions could increase inequity, especially if they require access to the internet. Internet-based interventions should consider potential risks for participants and incorporate skill-building sessions on safe internet and phone use.

## Introduction

There is a growing interest in the use of mobile phones to help young people (YP) access health information, and to support their engagement with health services. Data on YP’s use of information and communication technology (ICT), including mobile phones, are limited, particularly in low-income countries [1]. Such information is needed to inform the development of feasible and equitable digital health interventions. Data suggest that gender and socioeconomic gaps in access still exist in many countries [1], and there is a risk that the introduction of digital health interventions may increase inequity in access to health information and services. The importance of enhancing the use of enabling technology to promote sustainable development, gender equality, and the empowerment of all women and girls has been recognized in Sustainable Development Goals 17 and 5 [2].

The 2015 Zimbabwean Demographic and Health Survey found that 87% of households owned a mobile phone [3]. However, there is little quantitative data on YP’s access to ICT, for example, mobile phones, their patterns of use of ICT, and whether confidentiality would be a concern when communicating via phone or internet on sensitive topics. In particular, while it is widely believed that mobile phone use among YP is high, the functionality of the phones that are being used, preferences for platforms and apps, and the extent of potential challenges to intervention uptake such as confidentiality, cost, internet coverage or speed remain unknown.

With one-fifth of the Zimbabwean population aged between 15 and 24 years, YP’s health is central to the country’s development [4]. However, health service uptake by YP lags behind need. In this high HIV prevalence setting approximately half of all HIV-positive 15-24-year olds are unaware of their status [5] and use of preventive services such as contraception and voluntary male circumcision fall below national targets [6].

The aim of this study was to collect data on YP’s use of information and communication technology to inform the feasibility of implementing technology-based adolescent-health interventions in Zimbabwe.

## Methods

### Recruitment

A cross-sectional population-based survey was conducted from October to December 2018 in 3 urban communities (A, B, and C) in Harare province and 2 peri-urban communities (D and E) in Mashonaland East province. These 5 communities participated in formative work for the ongoing CHIEDZA sexual and reproductive health services intervention trial. The communities had been purposively selected to represent the urban and peri-urban communities that would be included in the trial. The survey was conducted in these communities so that the survey team could benefit from the existing research infrastructure and stakeholder relationships. Eligible participants were aged 13-24 years, resident in the study community at the time of the survey, and either provided informed consent (16-24 years) or provided assent with guardian consent (age 13-15 years).

We estimated that the prevalence of mobile phone (Textbox 1) ownership among 13-24-year olds would be 50%. Assuming 10% nonresponse and a design effect of 2 [3], a sample size of 686 YP would provide ±8% precision around this estimate. Using stratified sampling we aimed to recruit 60.1% (412/686) of participants from Harare, and 39.9% (274/686) of participants from Mashonaland East. A simple random sample of 100 GPS coordinates (primary sampling unit) was sampled per cluster from all potential points in the study areas using ArcGIS software version 10.5 (Esri). Points were randomly ordered and then sequentially visited by a team of interviewers. All households with front doors within 20 m of the sampled GPS point were visited. The household head was interviewed to obtain basic demographic information about the household and to obtain consent to interview any eligible YP. If the household head was not available, another household member aged 16+ years or a neighbor was asked to provide information on the composition of the household. Households with YP were visited a further two times in order to interview the household head. All YP in the selected household were eligible for recruitment.

**Definition of terms used in this paper.**

**Household:** A person or a group of related and unrelated persons who live together in the same dwelling unit(s), acknowledge 1 adult male or female as the head of the household, share the same housekeeping arrangements, and are considered a single unit. Household members were defined as individuals who have lived or intended to live in the household for 1 or more months, including school children regularly in residence during the school year [3].

**Internet access:** A person was considered to have internet access if they reported accessing the internet once or more in the last 3 months, including on a device belonging to a family member or employer (International indicator HH7) [7].

**Basic phone:** Mobile phone with limited features (no web browser or apps) that is used primarily for phone calls and sending SMS text messages.

**Feature phone:** Mobile phone with more features than a basic phone and usually has a camera, supports some apps but not all third-party apps, and features a web browser.

**Smartphone:** Mobile phone built on a mobile computing platform (eg, Apple OS, Android) and supports third-party apps.

**Phone ownership:** Has sole ownership of a mobile phone.

**Phone sharing:** Having joint ownership of or access to someone else’s mobile phone.

**Primary phone:** The phone that the respondent reports as the main phone that they use.

**Technological devices:** Desktop computer, laptop computer, tablet/iPad, mobile phone, iPod or other MP3 player, TV, radio, digital camera, gaming console, handheld gaming device.

### Data Collection

Participants responded to a 30-minute audio computer-assisted self-interviewing (ACASI) tablet-based questionnaire (Multimedia Appendix 1). ACASI was facilitated by trained research assistants who oriented the interviewees and were present during the interview to troubleshoot or answer questions. Questionnaire topics included use and ownership of technological devices including mobile phone, and access to and use of the internet. Questions were adapted from pre-existing questionnaires [8-11]. The questionnaire was developed in English and translated into Shona (the local language). Modifications were made to the questionnaire following pretesting with the study team and following the pilot survey which was conducted outside the selected study sites.

### Data Management and Analysis

The primary outcome was the prevalence of mobile phone ownership among 13-24-year olds. Secondary outcomes were the characteristics of mobile phones and phone use behavior; internet access and use behaviors; and ownership and use of other technological devices such as tablets, desktops, and laptops. Data were collected and recorded using Open Data Kit survey software with built-in logical checks and skip patterns on Android tablets. Data were analyzed using STATA version 15.1 (StataCorp). Using sampling weights and robust standard errors to account for the clustered sampling design (one-stage cluster sampling). Multivariable logistic regression was used to calculate age-adjusted odds ratios for the association between explanatory factors and mobile phone ownership, and with internet use. Potential explanatory variables were age, sex, marital status, community of residence, highest level of school attended, current occupational status, religion, travel for at least 1 month in past 12 months, length of time living in the community, and orphan status, with age being considered an a priori potential confounder. Wald tests adjusted for the clustered sampling design were used at each step of the analysis.

### Ethical Considerations

Ethical approval was obtained from the Institutional Review Board of the Biomedical Research and Training Institute (AP149/2018), the Medical Research Council of Zimbabwe (MRCZ/A/2362), and the London School of Hygiene and Tropical Medicine Research Ethics Committee (LSHTM REC, No. 15919). Written informed consent was obtained from parents or guardians of study participants aged below 16 years, along with participant assent. Participants aged 16 years and older consented independently.

## Results

### Study Population

**Figure 1:**
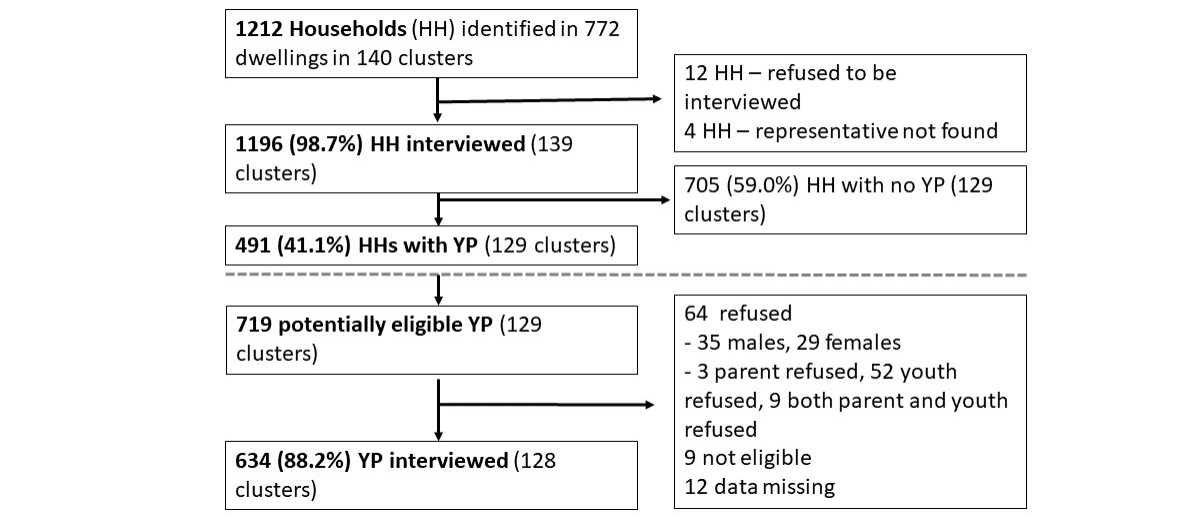
Survey recruitment (HH Household, YP young people).

In total, 1212 households were sampled from 140 GPS point clusters in 5 suburbs (A 25, B 48, C 21, D 42, and E 4; Figure 1). A total of 719 YP in the target age range were identified from 491 households (41.05% [491/1196] of successfully interviewed households); 634/719 (88.2%) YP were included in the study with 633/634 providing information on mobile phone ownership. Fewer GPS point clusters in community E were visited and only 10 participants were interviewed in that community. Community E was included in the descriptive analysis but excluded from regression analysis.

The mean age of the 634 participants was 18.0 years (SD 3.3) and 62.6% (397/634) were female. The majority (83.9%, 532/634) had never been married, 86.8% (550/634) had attended secondary school or higher, and only 14.7% (51/346) of out-of-school participants reported that they were working. The majority were Christian, had lived in the study community for at least 5 years, and had not traveled for at least 1 month in the past 12 months. Approximately one-third of respondents reported that one or both of their parents were dead or that a parent’s location was unknown (Table 1).

**Table 1:** Demographic characteristics of the study population (N=634).

Demographic characteristic		Sex of respondent		
		Male (N=237, 37.4%)	Female (N=397, 62.6%)	Total (N=634)
**Age group (years), n (%)**				
	13-15	65 (27.4)	96 (24.2)	161 (25.4)
	16-17	47 (19.8)	74 (18.6)	121 (19.1)
	18-19	55 (23.2)	89 (22.4)	144 (22.7)
	20-24	70 (29.5)	138 (34.8)	208 (32.8)
Mean age (years), mean (95% CI)		17.8 (17.4-18.2)	18.2 (17.8-18.5)	18.0 (17.8-18.3)
**Marital status, n (%)**				
	Married	5 (2.1)	64 (16.1)	69 (10.9)
	Cohabiting	1 (0.4)	15 (3.8)	16 (2.5)
	Never married	225 (94.9)	307 (77.3)	532 (83.9)
	Divorced/separated	6 (2.5)	11 (2.8)	17 (2.7)
**Highest level of school attended, n (%)**				
	Primary	32 (13.5)	50 (12.6)	82 (12.9)
	Secondary	195 (82.3)	330 (83.1)	525 (82.8)
	Higher (Tertiary)	9 (3.8)	16 (4.0)	25 (3.9)
	Never been to school	1 (0.4)	1 (0.3)	2 (0.3)
**Current occupational status, n (%)**				
	In school/university	121 (51.1)	167 (42.1)	288 (45.4)
	Out of school (working)	22 (9.3)	29 (7.3)	51 (8.0)
	Out of school (not working)	94 (39.7)	201 (50.6)	295 (46.5)
**Religion^a^, n (%)**				
	Roman Catholic	27 (11.5)	38 (9.6)	65 (10.3)
	Protestant	55 (23.5)	102 (25.7)	157 (24.9)
	Pentecostal	96 (41.0)	169 (42.6)	265 (42.0)
	Apostolic sect	19 (8.1)	67 (16.9)	86 (13.6)
	Other Christian/Muslim/Other	5 (2.1)	3 (0.8)	8 (1.3)
	No religion	32 (13.7)	18 (4.5)	50 (7.9)
**Traveled for at least 1 month in past 12 months, n (%)**				
	No	170 (71.7)	282 (71.0)	452 (71.3)
	Yes	67 (28.3)	115 (29.0)	182 (28.7)
**How long lived in community?^b^, n (%)**				
	<1 year	29 (12.3)	83 (20.9)	112 (17.7)
	1-4 years	44 (18.7)	98 (24.7)	142 (22.5)
	5+ years	162 (68.9)	216 (54.4)	378 (59.8)
**Orphan status, n (%)**				
	Double orphan	23 (9.7)	40 (10.1)	63 (9.9)
	Mother dead, father alive	21 (8.9)	31 (7.8)	52 (8.2)
	Mother alive, father dead, or unknown	40 (16.9)	70 (17.6)	110 (17.4)
	Both parents alive	153 (64.6)	256 (64.5)	409 (64.5)

^a^n=3 no response (men only).

^b^n=2 do not know (men only).

### Mobile Phone Ownership and Access

The prevalence of mobile phone ownership was 62.6% (396/633; 95% CI 58.5-66.5). Among the 237 who did not own a phone, 27 (11.4%) reported that they shared a phone (Table 2). In total, 423 out of the 633 YP interviewed (66.8%; 95% CI 62.3, 71.1) reported either owning or sharing a phone, with 18.0% (76/423) currently using (owning or sharing) 2 or more phones (Multimedia Appendix 2). The use of multiple phone numbers was common, with 26.5% (112/423) currently using and 42.8% (181/423) having used more than 1 phone number in the past year. The majority (85.3%, 361/423) of primary phones, either owned or shared, were reported to be smartphones.

Female phone sharers reported sharing phones with their mother (18/50, 36%), partner/boyfriend (14/50, 28%), or siblings (12/50, 24%), whereas male phone sharers reported sharing phones primarily with their siblings (11/20, 55%). Almost all (67/70, 96%) respondents who reported sharing phones did so at least once a week (Multimedia Appendix 2).

**Table 2:** Prevalence of phone ownership and phone sharing.

		Shares a phone					
		No		Yes		Total^a^	
Owns a phone		n (%)	95% CI	n (%)	95% CI	n (%)	95% CI
No		210 (33.2)	29.0-37.7	27 (4.3)	2.6-6.9	237 (37.4)	33.5-41.5
Yes		353 (55.8)	51.2-60.2	43 (6.8)	4.6-9.9	396 (62.6)	58.5-66.5
Total		563 (88.9)	85.0-91.9	70 (11.1)	8.1-15.0	633	Not applicable

^a^One participant did not respond to the question “Do you have or use a mobile phone?”

The main reasons for not owning or sharing a phone were have/had a phone but it is not working (27.6%, 58/210), cost (21.9%, 46/210), and not being allowed (17.1%, 36/210). However, 63.8% (134/210) of those who did not have access to a phone reported planning to buy one in the near future (Multimedia Appendix 3).

The median age at first mobile phone use was 13 years (IQR 12-15) and 15 years (IQR 13-16) for male and female respondents, respectively (Multimedia Appendix 4). First phones were primarily purchased by parents (237/367, 64.6%) or other relatives (83/367, 22.6%).

Prevalence of phone ownership increased with age of the respondent with 27.0% (43/159) of 13-15-year olds, 61.0% (72/118) of 16-17-year olds, 71.5% (103/144) of 18-19-year olds, and 84.7% (171/202) of 20-24-year olds owning a phone (OR 1.4, 95% CI 1.3-1.5) for each year increase (*P*<.001). In age-adjusted analysis there was weak evidence that mobile phone ownership was higher in those with at least secondary level education compared to those with primary or no education (secondary OR 2.3, 95% 1.1-4.8; tertiary OR 2.6, 95% 0.6-11.9; *P*=.09). The prevalence of mobile phone ownership was similar among male (153/236, 64.8%; 95% CI 57.8-71.2) and among female respondents (236/387, 61.0%; 95% CI 55.6-66.1; age-adjusted OR 0.7, 95% CI 0.5-1.1; *P*=.11; Table 3).

**Table 3:** Factors associated with phone ownership (N=623).

Factors		n	Prevalence, %	Unadjusted odds ratio (95% CI)	Age-adjusted odds ratio (95% CI)
**Age group (years)**				*P*<.001	
	13-15	159	27.0		
	16-17	118	61.0		
	18-19	144	71.5		
	20-24	202	84.7		
Per-year increase				1.40 (1.30-1.52)	
**Gender**				*P*=.39	*P*=.11
	Male	236	64.8	1^b^	1
	Female	387	61.0	0.85 (0.58-1.24)	0.73 (0.49-1.07)
**Marital status**				*P*<.001	*P*=0.13
	Married/cohabiting	79	79.8	1	1
	Never married	527	59.4	0.37 (0.21-0.64)	1.67 (0.87-3.18)
	Divorced/separated	17	76.5	0.83 (0.22-3.12)	0.64 (0.15-2.77)
**Religion**				*P*=0.13	*P*=0.10
	Roman Catholic	64	75.0	1	1
	Protestant	153	64.7	0.61 (0.31-1.22)	0.68 (0.33-1.40)
	Pentecostal	264	60.2	0.50 (0.28-0.92)	0.53 (0.28-1.00)
	Apostolic sect	85	56.5	0.43 (0.20-0.92)	0.46 (0.21-0.99)
	Other Christian/Muslim/Other	7	71.4	0.83 (0.17-4.05)	1.06 (0.35-3.20)
	No religion	47	57.5	0.45 (0.21-0.95)	0.42 (0.17-1.03)
**Community**				*P*=.37	*P*=.19
	A	178	59.0	1	1
	B	140	67.1	1.42 (0.89-2.28)	1.51 (0.91-2.52)
	C	147	59.2	1.01 (0.67-1.52)	0.97 (0.60-1.57)
	D	158	65.2	1.30 (0.79-2.16)	1.49 (0.82-2.69)
**Highest level of school attended**				*P*<.001	*P*=.09
	None/Primary	82	25.6	1	1
	Secondary	516	67.1	5.91 (3.22-10.86)	2.27 (1.08-4.77)
	Higher (tertiary)	25	88.0	21.30 (5.44-83.41)	2.64 (0.58-11.94)
**Current occupational status**				*P*<.001	*P*=.30
	In school/university	286	47.2	1	1
	Out of school (working)	51	90.2	10.29 (3.97-26.66)	2.24 (0.78-6.45)
	Out of school (not working)	286	72.7	2.98 (2.06-4.32)	1.09 (0.70-1.69)
**Traveled for at least 1 month in the past 12 months**				*P*=.41	*P*=.63
	No	443	61.4	1	1
	Yes	180	65.0	1.17 (0.81-1.69)	1.11 (0.72-1.71)
**How long lived in community?**				*P*=.08	*P*=.32
	<1 year	112	63.4	1	1
	1-4 years	137	70.8	1.40 (0.79-2.48)	1.51 (0.82-2.76)
	5+ years	372	59.1	0.84 (0.50-1.39)	1.05 (0.64-1.71)
**Orphan status**				*P*=.05	*P*=.12
	Double orphan	61	62.3	1	1
	Mother dead, father alive	52	75.0	1.82 (0.85-3.88)	1.96 (0.84-4.56)
	Mother alive, father dead or unknown	107	70.1	1.42 (0.83-2.44)	1.92 (1.03-3.60)
	Both parents alive	403	58.8	0.86 (0.50-1.50)	1.89 (1.07-3.34)

^a^n=623 as excludes 10 participants who were interviewed in community E.

^b^Reference.

### Phone Use Behavior

In total 4 in 10 phone owners reported that they never turned their phone off and 25.9% (103/397) reported that they could not do without their phone for a day. Among school-going phone-using respondents, just over half reported regularly bringing their phone to school. YP reported that the best thing about having a mobile phone was that it was convenient and made life easier (Multimedia Appendix 5).

The majority (280/423, 66.2%) of phone users spent US $1-3 per week on phone credit with 9.9% (42/423) spending nothing. Most phone users reported spending less on airtime in the past week when compared to other personal expenditure but 23.9% (101/423) reported having spent more on airtime. Phone credit was paid for by a combination of the respondent, their family members, or their friends. Half of females and a quarter of males reported that their boyfriend or girlfriend paid for phone credit (Multimedia Appendix 6).

**Figure 2:**
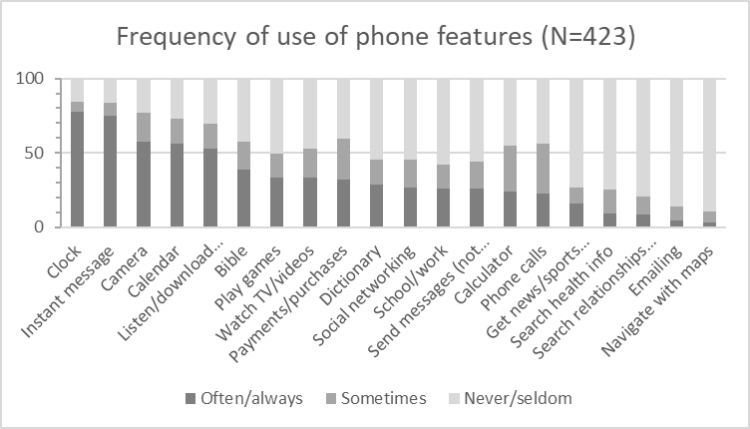
Frequency of use of different phone features among phone users.

The most commonly used phone features were the clock, instant messaging/chat, camera, and the calendar (Figure 2). The most commonly used app was WhatsApp (70.9%, 300/423). Other commonly used apps were Facebook, Facebook Messenger, internet browser, Instagram, Twitter, YouTube, dictionary, bible, and calculator (Figure 2, Multimedia Appendix 7). As many as 67.1% (108/161) of male and 56.9% (149/262) of female phone users reported playing games on their mobile phone. Candy Crush and Temple Run were the most popular games among females and FIFA, Temple Run, and Dream League were the most popular games among males.

Among phone users, a quarter report at least sometimes searching for health information and 20.3% (86/423) for information on relationships (Multimedia Appendix 7). A minority (16.3%, 69/423) report having ever used their phone to track their health. Males were more likely than females to report having used a phone to track their health (21.1% [34/161] versus 13.4% [35/262], *P*=.03). When asked to list the apps that they used to track their personal health, the majority mentioned web browsers, YouTube, and social media platforms. Specific apps mentioned related to fitness, blood pressure, sugar levels, body temperature, HIV testing, skincare, period trackers, home remedies app, and health tips (including daily health tips).

In total, 4 in 10 phone owners (44.2%, 175/396) reported that the information stored on their phone was not private. A similar proportion thought that the information they sent on their phone (40.4%, 160/396) and received on their phone (40.9%, 162/396) was not private. Only two-thirds (62.1%, 246/396) had a password to lock/unlock their phone and 17.7% (70/396) had passwords for any apps on their phone (Table 4).

**Table 4:** Security and privacy associated with phone use among phone owners (N=396).

Questions on security and privacy		n (%)	95% CI
**How private do you consider the information that you send when using a phone?**			
	Very private	148 (37.4)	29.4-46.1
	Somewhat private	88 (22.2)	17.6-27.7
	Not private	160 (40.4)	33.5-47.8
**How private do you consider the information that you receive when using a phone?**			
	Very private	145 (36.6)	29.3-44.7
	Somewhat private	89 (22.5)	18.1-27.6
	Not private	162 (40.9)	33.8-48.4
**How private do you consider the information stored on your phone?**			
	Very private	146 (36.9)	29.8-44.6
	Somewhat private	75 (18.9)	14.7-24.1
	Not private	175 (44.2)	37.0-51.7
**Do you have passwords to lock/unlock your phone?**			
	No	150 (37.9)	33.5-42.4
	Yes	246 (62.1)	57.6-66.5
**Do you have passwords for any apps on your phone?**			
	No	326 (82.3)	78.4-85.6
	Yes	70 (17.7)	14.4-21.6

### Internet Use

A total of 407/631 (64.5%) respondents (95% CI 60.3-68.5) had access to the internet (used in the last 3 months), with 73.8% (468/634) reporting ever using the internet. Ever internet users reported accessing the internet frequently (at least once per week) on a mobile phone (78.2%, 366/468) or on a computer at work or school (18.6%, 87/468). Frequent access to the internet on other computers was rare: commercial internet outlet (5.6%, 26/468), at home (6.8%, 32/468), at someone else’s house (4.3%, 20/468), or in a library/community facility (4.1%, 19/468; Multimedia Appendix 8).

Internet access in the last 3 months increased with age (OR 1.2, 95% CI 1.2-1.3, per year increase; *P*<.001). In age-adjusted analysis, internet access was lower among females (adjusted OR 0.5, 95% CI 0.4-0.8; *P*=.001). Internet access was higher among the never married compared to the married and cohabitating (adjusted OR 2.8, 95% CI 1.5-5.5; *P*=.001), among those who had secondary education compared to primary or no education (adjusted OR 2.3, 95% CI 1.2-4.2; *P*=.03), and among those who had lived in the community for more than 1 year (*P*=.003; Table 5). Mobile phone owners had 9 times the odds of having access to the internet compared to nonphone owners (adjusted OR 8.7, 95% CI 5.6-13.5; *P*<.001). There was no evidence of a difference in internet access according to religion, community, travel in the past 12 months, or orphan status.

**Table 5:** Factors associated with internet access (N=621)^a^.

Factors		n	Prevalence, %	Unadjusted odds ratio (95% CI)	Age-adjusted odds ratio (95% CI)
**Age group (years)**				*P*<.001	
	13-15	156	37.8		
	16-17	119	69.8		
	18-19	144	73.6		
	20-24	202	77.2		
Per year increase				1.22 (1.15-1.30)	
**Gender**				*P*=.007	*P*=.001
	Male	234	72.7	1^b^	1
	Female	387	60.5	0.58 (0.39-0.86)	0.52 (0.35-0.76)
**Marital status**				*P*=.34	*P*=.01
	Married/cohabiting	79	65.8	1	1
	Never married	525	64.4	0.94 (0.57-1.55)	2.83 (1.45-5.53)
	Divorced/separated	17	82.4	2.42 (0.57-10.37)	2.19 (0.49-9.82)
**Religion**				*P*=.11	*P*=.13
	Roman Catholic	63	77.8	1	1
	Protestant	153	68.0	0.61 (0.30-1.24)	0.67 (0.31-1.45)
	Pentecostal	264	64.4	0.52 (0.25-1.06)	0.56 (0.26-1.19)
	Apostolic sect	84	53.6	0.33 (0.15-0.71)	0.35 (0.15-0.79)
	Other Christian/Muslim/Other	8	62.5	0.48 (0.10-2.17)	0.56 (0.15-2.10)
	No religion	46	60.9	0.44 (0.18-1.08)	0.44 (0.18-1.09)
**Community**				*P*=.06	*P*=.05
	A	177	63.3	1	1
	B	139	77.0	1.94 (1.07-3.51)	2.03 (1.11-3.71)
	C	147	61.9	0.94 (0.61-1.46)	0.92 (0.58-1.47)
	D	158	59.5	0.85 (0.53-1.38)	0.87 (0.51-1.50)
**Highest level of school attended**				*P*<.001	*P*=.03
	None/Primary	82	35.4	1	1
	Secondary	514	68.9	4.04 (2.40-6.82)	2.28 (1.23-4.21)
	Higher (Tertiary)	25	84.0	9.59 (3.05-30.18)	2.99 (0.89-10.08)
**Current occupational status**				*P*=.009	*P*=.37
	In school/university	285	58.3	1	1
	Out of school (working)	51	80.4	2.94 (1.36-6.33)	0.90 (0.38-2.16)
	Out of school (not working)	285	69.1	1.60 (1.07-2.41)	0.72 (0.45-1.17)
**Traveled for at least 1 month in the past 12 months**				*P*=.25	*P*=.32
	No	442	63.6	1	1
	Yes	179	68.7	1.26 (0.85-1.87)	1.23 (0.82-1.85)
**How long lived in community?**				*P*=.01	*P*=.003
	<1 year	110	51.8	1	1
	1-4 years	137	69.3	2.10 (1.25-3.55)	2.30 (1.29-4.12)
	5+ years	372	67.5	1.93 (1.19-3.13)	2.50 (1.47-4.24)
**Orphan status**				*P*=.74	*P*=.28
	Double orphan	61	62.3	1	1
	Mother dead, father alive	52	67.3	1.25 (0.59-2.64)	1.24 (0.55-2.79)
	Mother alive, father dead, or unknown	108	68.5	1.32 (0.69-2.52)	1.55 (0.75-3.19)
	Both parents alive	400	64.3	1.09 (0.60-1.98)	1.79 (0.94-3.41)
**Owns a phone**				*P*<.001	*P*<.001
	No	232	33.6	1	1
	Yes	388	84.0	10.38 (6.84-15.75)	8.67 (5.58-13.47)

^a^10 respondents from community E were excluded and 3 respondents did not provide information on the timing of most recent internet access.

^b^Reference.

The most common technological devices that respondents had at home were televisions (89.1%, 565/634), mobile phones (87.5%, 555/634), and radios (71.1%, 451/634). Ever use of technological devices was higher than household ownership but showed similar patterns with a high proportion reporting ever use of televisions, mobile phones, and radios. Over half of respondents had ever used a desktop computer (61.7%, 391/634) and laptop computer (65.6%, 416/634; Multimedia Appendix 9).

YP reported that the thing that they most disliked about the internet was seeing unwanted sexual content (29.2%, 136/465) and violent stories, photos, and videos (13.1%, 61/465). The most common suggestions on how to make the internet better were cheaper data plans (36.8%, 171/465), making access to mobile phones and computers easier (14.6%, 68/465), better internet coverage (12.9%, 60/465), and high-speed connectivity (12.7%, 59/465; Multimedia Appendix 10).

## Discussion

### Principal Findings

YP in Harare and Mashonaland East had high levels of phone ownership and internet access and access increased with age. A minority of YP used their phones to seek health information or to support their health. Challenges that YP face when using mobile phones and the internet include the cost of data, access to phones/computers, speed of connection, exposure to unwanted sexual and violent content, and concerns about security and confidentiality. Older age groups could be targeted for phone-based interventions but ensuring equitable access to data and charging facilities as well as training on safe internet use are necessary.

### Limitations

This study only collected data on YP living in urban and peri-urban areas. An estimated 68% of Zimbabwe’s population live in rural areas [12] and there is a well-documented digital divide with internet use much lower in rural compared to urban areas [13-15]. Data on contextual factors, such as the availability of electricity to charge devices, were not collected. A relatively high proportion of respondents reported access to a smartphone but we did not collect data on the functionality of the phones. We gathered limited information on respondents’ current use of their phone to access health information and services. In-depth qualitative studies are needed to better understand their current use, including barriers and facilitators, and to explore willingness to use their phones to access information and services. Alternative ways to understand YP’s digital lives, which could be considered for future studies, are the use of diaries or qualitative interviews which can probe for a detailed understanding of use throughout the day [16,17].

### Comparison With Prior Work

This study provides a unique insight into phone ownership and use among YP in Zimbabwe as there is little published data available for this population. Phone ownership among 20-24-year olds in this study (84.7%, 171/202) was higher than national estimates from the most recent 2015 Zimbabwean DHS (71.1% among females and 76.5% among males) [18], but phone access was in line with levels seen in higher-income Sub-Saharan African (SSA) countries such as South Africa. A 2017 survey among adults aged over 18 in 6 African countries (excluding Zimbabwe) found that 72%-93% of 18-29-year olds reported owning a mobile phone, with the proportion owning smartphones ranging from 17% in Tanzania to 63% in South Africa [19]. A 2013/14 household survey among 9-18-year olds in South Africa, Ghana, and Malawi also found a large variation in phone ownership ranging from 6.2% in females in Malawi to 50.9% among males in South Africa [20,21]. A 2012 survey of secondary school students in South Africa found that 81.1% owned or had access to a mobile phone [14].

Although phone ownership was comparable between genders, males reported increased internet access/usage. Phone sharing was relatively common with differences in phone sharing between males and females, that is, who they share with. The lack of an association between gender and phone ownership observed in this study is consistent with findings from South Africa [20] and there is some evidence to suggest that as the prevalence of phone ownership increases, the gender divide decreases [22]. By contrast, we found higher access to the internet among males, those not currently married, those with greater than primary education, and longer-term residents. Observed gender differences in internet access are in keeping with other studies from SSA. A 2018 multicountry study among adolescents in low- and middle-income countries found that boys were more likely than girls to have smartphones (through which they can access the internet) and used a wider variety of phone features compared to girls [23]. A Ugandan study among 18-24-year olds found high phone ownership among both sexes but lower internet use among females compared to males [24]. The 2019 GSMA mobile gender report also found a bigger gap for internet use than phone use, with women also using a smaller range of services and spending less on their phones than men [25]. Global goals have been set to provide internet access for everyone and access to digital technology is considered an important component to help adolescents achieve their rights [26-28]. The findings from this study contribute to our understanding of which YP have and do not have access to phones and the internet.

### Implications for Interventions

Some kinds of phone-based interventions are likely to be feasible in this population and phones are increasingly being used in Zimbabwe to facilitate health information and service delivery [29-31]. A quarter of YP in this study reported sometimes using their phones to access health information or services, suggesting that phones may be an acceptable medium for health information and services. A study of informal mHealth (mobile health) in South Africa, Ghana, and Malawi found that almost a fifth of YP surveyed reported using their phones in the previous year to obtain health advice or information [21]. Similarly, across 7 SSA countries an average of 17% of mobile phone owners reported having used their phone in the past 12 months to get information about health and medicine [32].

Current phone use behaviors and preferences can inform the kind of intervention that might be attractive to YP. Many of the interviewed YP use games and social media, and so health interventions that incorporate gaming and social aspects may be attractive. A qualitative study in the United States identified 3 reasons that adolescents 13-18 years reported using ICT for their health: to gather information, to share experiences and view others’ experiences in order to gain social support or inspiration, and to track health behaviors and goals [33]. In this study participants reported using features that would correspond with each of these 3 reasons (eg, used web browsers, social media platforms, and health monitoring apps).

Older age groups could be targeted for phone-based interventions but some issues require consideration when planning mobile phone interventions with YP in this setting.

#### Access and Feasibility

Careful consideration needs to be given to equitable access to interventions, especially in terms of age and gender. Internet-based interventions that require access to a smartphone or computer may be less feasible and increase inequality. Cost, internet coverage, and speed may hinder intervention uptake [23,25]. However, as with access to mobile phones, these factors are likely to change over time. The functionality of phones and the prevalence of fake smartphones should be explored during formative work as not all smartphones may be capable of running additional apps. Recording whether participants have multiple phones or phone numbers may improve follow-up [34]. Phone sharing is relatively common and YP’s access to phones may be controlled by someone else which may limit their access and raises the issue of confidentiality [22,35]. In this study females were most likely to share phones with their mothers and boyfriends/partners, and boys with their siblings. Females also reported primarily sharing phones with their mothers in other SSA countries [23]. Phone sharing behaviors coupled with low confidence in the security of information on phones may lead to poor uptake of interventions. This may be particularly important if phones are to be used to deliver information or interventions on sensitive topics such as sexual health. While the use of shared phones for sexual health interventions could be harmful for the participant, it could equally result in healthy discussions between the participant and phone owner.

#### Safety and Skills

Whether literacy and technical skills are a barrier to phone and internet use should be assessed, and if so then additional training and support provided [25]. Potential risks associated with phone-based interventions need to be considered when designing interventions and monitored closely during implementation. One potential risk of internet use is exposure to unwanted content [27]. Online risk can be categorized as exposure to “content,” “contact” (where the YP participates, even unwillingly), or “conduct” (where the YP is the actor) [14]. In this study YP report exposure to content but they did not mention, nor did we specifically probe about other risks. Research suggests that YP are often resilient and have mechanisms to cope with these risks but that those who are vulnerable offline are often vulnerable online [14]. YP in this study reported low levels of phone security and confidence in the privacy of information. Safety and security concerns can be barriers to mobile phone use [25]. Training for YP should include security and confidentiality (eg, use of passwords), and the development of resilience to navigate risk in the online environment [28].

### Conclusions

Mobile phone–based health interventions may be feasible for urban and peri-urban Zimbabwean YP. However, interventions could increase inequity especially if they require access to the internet. Provision of free internet access may remove this inequity, but additional factors such as capability to recharge one’s mobile phone and the technological capabilities of phones should be taken into account. Involving YP from the target communities in intervention design teams is recommended to develop more appropriate and feasible interventions. Potential risks to intervention participants should be closely monitored and mitigated against by incorporating skill-building sessions on safe internet and phone use into recruitment activities.

## Appendix

Multimedia Appendix 1 Young people's questionnaire.

Multimedia Appendix 2 Characteristics of phones and phone sharing (among n=423 phone users, 396 phone owners, 70 phone sharers).

Multimedia Appendix 3 Non-phone owners.

Multimedia Appendix 4 First phone.

Multimedia Appendix 5 Frequency of phone use and attitudes towards phone.

Multimedia Appendix 6 Expenditure on phone.

Multimedia Appendix 7 Frequency of use of different features (row %; among n=423 phone users).

Multimedia Appendix 8 How often use the internet at different locations? (among n=468 who have ever used the internet).

Multimedia Appendix 9 Household ownership of other technological devices (n=634).

Multimedia Appendix 10 Attitudes to the internet (among n=465 who have ever used the interneta).

## Abbreviations

ACASI: audio computer-assisted self-interviewing
ICT: information and communication technology
SSA: sub-Saharan Africa
YP: young people
