# Supplementary material for: Mobile Phone Access and Implications for Digital Health Interventions Among Adolescents and Young Adults in Zimbabwe: Cross-Sectional Survey
Source: JMIR Mhealth Uhealth. 2021 Jan 13;9(1):e21244. doi: 10.2196/21244 (PMC7840276; doi:10.2196/21244)
Supplement: Multimedia Appendix 1 [file mhealth_v9i1e21244_app1.docx]

**Young People’s use of technology, and mental health and well-being (YTM) study**

**Young People’s questionnaire**

| **Section 0- Recruitment** | | |
| --- | --- | --- |
| **Field** | **Question** | **Answer** |
| Community (required) | Select community | 1  2 |
| Community2 (required) | Select community AGAIN  Response constrained to . = ${community} | 1  2 |
| Location (required) | Select the LOCATION | 1 URBAN  2 SEMI URBAN |
| Dwell (required) | Enter the number of the structure  Response constrained to :. <500 |  |
| Dwell2 (required) | Enter the number of the structure AGAIN  Response constrained to:.=${struct} |  |
| hh (required) | Enter the household number  Response constrained to :. <40 |  |
| hh2 (required) | Entre the household number AGAIN  Response constrained to:. =${hh} |  |
| yp_id (required) | Enter the young person’s number  If this is the first young person that you are interviewing in this household, enter 1  Response constrained to: . <10 |  |
| yp_id2 (required) | Enter the young person’s’s number AGAIN  Response constrained to.=${team} |  |
| Interv (required) | Interviewer select your name |  |
| Interv2 ( required) | Interviewer select your name AGAIN  Response constrained to :.=${interv} |  |
| Lang1 (required) | Please select the language for this interview | 1 Shona  2 English |

| **SECTION 1- DEMOGRAPHIC PROFILE** | | |
| --- | --- | --- |
| Q101 Gender (required) | Q101 Respondent’s gender | 1 Male  2 Female |
| Q102 (required) | Q102 How old were you at your last birthday?  *Response constrained to :.>12 and .<25* | (years) |
| Q103 (required) | Q103 What is your current marital status? | 1. Married 2. Cohabiting/living with a man/woman as if married 3. Never married 4. Widow 5. Divorced/separated   99. No response |
| Q104 (required) | Q104 Have you ever attended school? | 1. Yes 2. No   97. Don’t know  99. No response |
| Q105 | Q105 Which of the following best describes your current occupational status? | 1. In-school (primary, secondary, college, university) 2. Out of school (working) 3. Out of school (not working) |
| Q106 (required) | Q106 What is the highest level of school you attended: primary, secondary, or higher?  Question relevant when: ${q104} = 1 or ${q104} = 99 | 1. Primary 2. Secondary 3. Higher (Tertiary) 4. No response 5. Don’t know |
| Q107 (required) | Q107 What is your religion? | 1. Traditional 2. Roman Catholic 3. Protestant 4. Pentecostal 5. Apostolic sect 6. Other Christian 7. Muslim 8. No religion 9. Other (specify) 10. Don’t know 11. No response |
| Q107_oth (required) | Q107_oth Others specify  Question relevant when: ${q107}=9  Response constrained to: string-length (.)>2 |  |
| Q108 (required) | Q108 Outside your home, what language do you speak the most? |  |
| Q109 (required) | Q109 How long have you been living in this community? | 1. Number of years 2. Number of months 3. Have always lived in this community   97. Don’t know  99. No response |
| Q109_years | Q109 Specify in YEARs  Question relevant when: ${q109} = 1  Response constrained to: (.>0 and .<= ${q102} ) |  |
| Q109_mths | Q109 Specify in MONTHs  Question relevant when: ${q109} = 2  Response constrained to: .>0 and .<12 |  |
| Q110 | Q110 In the last 12 months, have you stayed in a place other than this community for one month or more? | 1. Yes 2. No   97. Don’t know  99. No response |
| Q111 | Q111 In total approximately how long have you spent outside this community in the last 12 months?  This includes ALL the time spent outside the community, including for periods less than one month.  Record the response in WEEKS<br/><br/>If "DON'T KNOW" enter "9997" or If "NO RESPONSE" enter "9999"  Question relevant when: ${q110} = 1 or ${q110} = 99  Response constrained to: (.>3 and .<=52) or . =9999 or . =9997 |  |
| Q112 | Q112 Is your natural father alive? | 1. Yes 2. No 3. Don’t Know 4. No response |
| Q113 | Q113 Is your natural mother alive? | 1. Yes 2. No 3. Don’t Know 4. No response |

| **SECTION 2: USE OF TECHNOLOGY** | |  |
| --- | --- | --- |
| Q201 | Q201 Please tell us about some technological devices- which of the following have you ever used before, which did you use yesterday, and which ones do you have at home?  *A feature phone allows you to go on the internet but cannot download third party applications e.g. WhatsApp*  *A smart phone allows you to go on the internet and download applications e.g. WhatsApp* | \| **Device** \| **Ever used** \| **Used yesterday** \| **Have at home** \| \| --- \| --- \| --- \| --- \| \| Normal/’desktop’ computer \|  \|  \|  \| \| Laptop computer (including notebooks and netbooks) \|  \|  \|  \| \| Tablet/ iPad \|  \|  \|  \| \| Feature (non-smart) Cell phone \|  \|  \|  \| \| Smart cell phone \|  \|  \|  \| \| ipod or other MP3 player \|  \|  \|  \| \| TV \|  \|  \|  \| \| Radio \|  \|  \|  \| \| Digital camera \|  \|  \|  \| \| Gaming console (Playstation, Xbox, Nintendo wii etc.) \|  \|  \|  \| \| Handheld gaming device (e.g. DS, PSP, …) \|  \|  \|  \| \|  \|  \|  \|  \| |
| Q202 | Q202 Have you ever used the internet?  *Someone is using the internet when they are doing any of the following:*   - *Browsing websites (e.g. Google, Yahoo, Amazon, Wikipedia)* - *Visiting social networking sites (e.g. Facebook, Twitter, LinkedIn, Pinterest, YouTube, Instagram)* - *Sending e-mails of instant messages (e.g. via Facebook messenger, WhatsApp, KakaoTalk, LINE, Viber, Snapchat)* | 1. Yes 2. No **(skip to Q209)**   97. Don’t know **(skip to Q209)**  99 No response **(skip to Q209)** |

| Q203 | Q203 If yes, when is the last time that you used the internet? | 1. Today 2. Yesterday 3. Within the last seven days 4. Within the last month 5. In the last three months 6. Longer than three months 7. Unsure/do not know |
| --- | --- | --- |
| Q204 | Q204 When you use the internet, how often do you do so…. | \|  \| Several times/day \| Once/day \| At least once a week \| Less often \| Never \| DK \| \| --- \| --- \| --- \| --- \| --- \| --- \| --- \| \| On a computer at school/ work \|  \|  \|  \|  \|  \|  \| \| On a computer at home \|  \|  \|  \|  \|  \|  \| \| On a cell/mobile phone \|  \|  \|  \|  \|  \|  \| \| At a computer in the library/ other community internet facility \|  \|  \|  \|  \|  \|  \| \| At a commercial internet café- where you pay for the access \|  \|  \|  \|  \|  \|  \| \| At a computer in someone else’s house \|  \|  \|  \|  \|  \|  \| |
| Q205 | Q205 Roughly how much time do you spend on the internet when you go online? (select one) | 1. 5 minutes 2. 10 minutes 3. 15 minutes 4. 20 minutes 5. 25 minutes 6. 30 minutes 7. More than 30 minutes |

| Q206 | Q206 What do you like the most about the internet? | 1. I can easily find information 2. It helps with my school/college/university studies 3. I can communicate (send message/talk mostly 1:1) with my friends/relatives 4. I can communicate to lots of people at once (through social media, group messages on WhatsApp) 5. Entertainment (Playing games, listening to music, watching videos) 6. Education 7. Shopping 8. Looking for a job or career advice 9. Supporting your business ( eg advertising, contacting customers etc.) 10. Other (specify) |
| --- | --- | --- |
| Q207 | Q207 What do you dislike the most about the internet? | 1. I see violent stories, photos, videos 2. I see sexual content I don’t want to see 3. There is bullying to me and my friend 4. People share embarrassing things about me 5. Scams 6. There is nothing I dislike 7. Other (specify) |
| Q208 | Q208 What one change would you like to see so that use of the internet could be better for you? | 1. Better internet coverage 2. Cheaper data plans 3. Easier access to mobile phones and computers 4. Higher speed connectivity 5. Other (specify) |

| Q209 | | Do you have or use a mobile (cell ) phone? | | | | | 1. Yes 2. No   99. No response | | | |
| --- | --- | --- | --- | --- | --- | --- | --- | --- | --- | --- |
| Q209a | | If Yes, how many phones you own by yourself ? | | | | | ------------------------ | | | |
| Q209b | | If Yes, how many phones do share with someone else? | | | | | ------------------------- | | | |
| Q210 | | Q210 How many mobile phone numbers do you currently use? | | | | | | | | |
| Q211 | | Q211 How many different mobile phone numbers have you had in the past year including your current number (s)? | | | | | | | | |
| Q212 | | Q212 What phone(s) do you own or share?  Skip to Q214 if don’t report sharing a phone with anyone | | | | \| **Company** \| **Model** \| **Smart phone (Y/N)** \| **Year purchased** \| **Shared ownership (Y/N)** \| \| --- \| --- \| --- \| --- \| --- \| \| Nokia \|  \|  \|  \|  \| \| G-Tide \|  \|  \|  \|  \| \| Huawei \|  \|  \|  \|  \| \| Sony Ericsson \|  \|  \|  \|  \| \| Motorola \|  \|  \|  \|  \| \| Panasonic \|  \|  \|  \|  \| \| Samsung \|  \|  \|  \|  \| \| LG \|  \|  \|  \|  \| \| Siemens \|  \|  \|  \|  \| \| Apple iPhone \|  \|  \|  \|  \| \| Blackberry \|  \|  \|  \|  \| \| ZTE \|  \|  \|  \|  \| \| HTC \|  \|  \|  \|  \| \| Microsoft \|  \|  \|  \|  \| \| Lenovo \|  \|  \|  \|  \| \| Other \|  \|  \|  \|  \| | | | | |
| Q213 | | | Q213 Who do you share ownership of your phone with? | | | | | 1. Sister 2. Brother 3. Mother 4. Father 5. Other adult in household 6. Friend 7. Partner/boyfriend/girlfriend 8. Other (specify) | | |
| Q213_oth | | | Q213_oth Specify other | | | | |  | | |
| Q214 | | | Q214 Which mobile provider do you use normally? | | | | | 1. Econet 2. Telecel 3. Netone 4. Africom | | |
| Q215 | | | Q215 Do you use any other networks? (select all that apply) | | | | | 1. Econet 2. Telecel 3. Netone 4. Africom | | |
| Q216 | | | Q216 For your phone, does airtime need to be purchased in advance or is it paid monthly through a contract? | | | | | 1. Buy airtime 2. Pay monthly through a contract 3. Don’t know 4. No response | | |
| Q217 | | | Q217 How much airtime, in dollars, did you use in the past week? | | | | | 1. None 2. < $1 3. $1-3 4. $4 5. $5-7 6. $8 7. $9+   97. Don’t know  99 No response | | |
| Q218 | | | Q218 How much money did you spend on other things for yourself in the past week excluding airtime for cell phones? (money spend on food, clothing, going out…). If you don’t know the exact amount, please guess or give a rough estimate. | | | | | 1. None 2. < $1 3. $1-3 4. $4 ($1/week) 5. $5-7 6. $8 ($2/week) 7. $9+   97. Don’t know  99 No response | | |
| Q219 | | | Q219 Who usually pays for the credit (multiple responses possible)? | | | | | 1. I do 2. My parents/ legal guardians 3. Other relative 4. Friend 5. Boyfriend/girlfriend 6. I don’t spend money on airtime 7. Other (specify) 8. Don’t know 9. No response | | |
| Q220 | | | Q220 At what age did you start using a mobile phone? | | | | | (yrs)   1. Don’t know 2. No response | | |
| Q221 | | | Q221 Why did you get your first phone? | | | | | 1. Everyone around you had one 2. You wanted one 3. Somebody (friend/parents) asked you to get one for yourself 4. Passed on to me 5. Gifted to me 6. Extra set available 7. Business purpose 8. Convenience 9. Work required it 10. Other (specify) | | |
| Q222 | | | Q222 Who bought your first phone? | | | | | 1. I did 2. My parents 3. Other relative 4. Friend 5. Boyfriend/girlfriend 6. Company/work 7. Other (specify) 8. Don’t know 9. No response | | |
| Q222_oth | | | Q222_oth Specify other | | | | |  | | |
| Q223 | | | Q223 When is your phone usually switched off? (multiple options allowed) | | | | | 1. Never 2. When I am sleeping 3. When I am at school/ work 4. When I have run out of credit 5. When the battery is low 6. Other (specify) 7. Don’t know 8. No response | | |
| Q224 | | | Q224 How often do you place your phone on ‘SILENT’ or ‘VIBRATE’ mode? | | | | | 1. No, never 2. Seldom/rarely 3. Sometimes 4. Often 5. Yes, always | | |
| Q225 | | | Q225 Do you take your l to school ? | | | | | 1. Yes, every day 2. Yes, sometimes 3. No 4. Don’t know 5. No response | | |
| Q226 | | | Q226 Can you do without a mobile for a day? | | | | | 1. Yes 2. No 3. Don’t know   99 No response | | |
| Q227 | | | Q227 If No, why not? (specify) | | | | |  | | |
| Q228 | | | Q228 According to you, the best part of having a mobile phone is  **Proceed to Q237** | | | | | 1. It is stylish 2. It makes your life easier ie it is ‘convenient’ 3. You feel safer 4. Any other _________ | | |
| Q229 | | Q229 If you don’t have a mobile phone, kindly tell me why not? | | | | | | 1. It is not allowed 2. You don’t require it 3. It is costly 4. You don’t want to be contactable, i.e. you don’t want to be reached all the time 5. Other ____ | | |
| Q230 | | Q230 Do you have access to a phone? | | | | | | 1. Yes- smart phone 2. Yes- feature (non-smart) phone 3. No 4. No response | | |
| Q231 | | Q231 At what age did you start using a mobile phone? | | | | | | (yrs)   1. Don’t know 2. Not applicable   99 No response | | |
| Q232 | | Q232 How often do you have access to a phone? | | | | | | 1. More than once a day 2. Once a day 3. A few times aweek 4. Once a week 5. Less than once a week   99 No response | | |
| Q233 | | Q233 Do you always access the same phone? | | | | | | 1. Yes 2. No   99. No response | | |
| Q234 | | Q234 Who owns the phone that you access the most? | | | | | | 1. Sister 2. Brother 3. Mother 4. Father 5. Other adult in household 6. Friend 7. Partner/boyfriend/girlfriend 8. Other (specify) | | |
| Q234_oth | | Q234_oth Specify | | | | | |  | | |
| Q235 | | Q235 Are you planning to buy a mobile phone in near future? | | | | | | 1. Yes 2. No   97. Don’t know  99 No response | | |
| Q236 | | Q236 If yes, when? | | | 1. Within the next month (already in process) 2. Sometime in the next 3-6 months 3. Maybe when I start working/earning 4. Maybe when I start going to secondary school (?) | | | | | |
| Q237 | | Q237 How frequently do you use a mobile phone for: | | | \| **Function** \| **Always** \| **Often** \| **Sometimes** \| **Seldom/Rarely** \| **Never** \| \| --- \| --- \| --- \| --- \| --- \| --- \| \| Phone calls \|  \|  \|  \|  \|  \| \| Sending text or picture messages (not via an app) \|  \|  \|  \|  \|  \| \| Sending instant messages or chat services (e.g Whatsapp, Facebook etc.) \|  \|  \|  \|  \|  \| \| Playing games \|  \|  \|  \|  \|  \| \| Watch TV/movies/videos \|  \|  \|  \|  \|  \| \| Listen to/ download music/radio \|  \|  \|  \|  \|  \| \| Getting news updates (sports or other) \|  \|  \|  \|  \|  \| \| Calculator \|  \|  \|  \|  \|  \| \| Social networking sites e.g. twitter, Instagram, Facebook \|  \|  \|  \|  \|  \| \| Making payments/ purchasing goods \|  \|  \|  \|  \|  \| \| Camera \|  \|  \|  \|  \|  \| \| Navigating with maps \|  \|  \|  \|  \|  \| \| E-mailing \|  \|  \|  \|  \|  \| \| Research for school/work or doing homework /revision e.g. Ruzivo \|  \|  \|  \|  \|  \| \| Search for information about my health \|  \|  \|  \|  \|  \| \| Search for information about relationships and sex \|  \|  \|  \|  \|  \| \| Calendar \|  \|  \|  \|  \|  \| \| Clock \|  \|  \|  \|  \|  \| \| Dictionary \|  \|  \|  \|  \|  \| \| Bible \|  \|  \|  \|  \|  \| | | | | | |
| Q238 | Q238 What are the three apps that you most often use ? | | | | | | | | | 1. Facebook messenger 2. Facebook 3. WhatsApp 4. Twitter 5. Instagram 6. Pinterest 7. YouTube 8. Other (specify) |
| Q239 | Q239 What are the three games you most often play on a cell phone? | | | | | | | | | 1.  2.  3.  9. Don’t play games |
| Q240 | Q240 Have you ever used your phone for tracking your health? | | | | | | | | | 1. Yes 2. No |
| Q241 | Q241 Please list the applications that you use to track your personal health measures | | | | | | | | |  |
| Q242 | Q242 How ‘private’ do you consider the information that you send when using a phone? | | | | | | | | | 1. Very private 2. Somewhat private 3. Not private |
| Q243 | Q243 How ‘private’ do you consider the information that you receive when using a phone? | | | | | | | | | 1. Very private 2. Somewhat private 3. Not private |
| Q244 | Q244 How ‘private’ do you consider the information stored in your mobile phone? | | | | | | | | | 1. Very private 2. Somewhat private 3. Not private |
| Q245 | Q245 Do you have passwords to lock/unlock your phone? | | | | | | | | | 1. Yes 2. No   97. Don’t know  99 No response |
| Q246 | Q246 Do you have passwords for any applications on your phone? | | | | | | | | | 1. Yes 2. No   97. Don’t know  99 No response |
| Q247 | Q247 If yes, which applications have passwords? | | | | | | | | |  |
| **SECTION 3- MENTAL HEALTH – SSQ** | | | | | | | | | | |
|  | | | | In the past week….. | | | | |  | |
| Q301 | | | | There were times in which I was thinking deeply or thinking about many things | | | | | 1. Yes 2. No | |
| Q302 | | | | I found myself sometimes failing to concentrate | | | | | 1. Yes 2. No | |
| Q303 | | | | I lost my temper or got annoyed over trivial matters | | | | | 1. Yes 2. No | |
| Q304 | | | | I had nightmares or bad dreams | | | | | 1. Yes 2. No | |
| Q305 | | | | I sometimes saw or heard things which others could not see or hear | | | | | 1. Yes 2. No | |
| Q306 | | | | My stomach was aching | | | | | 1. Yes 2. No | |
| Q307 | | | | I was frightened by trivial things | | | | | 1. Yes 2. No | |
| Q308 | | | | I sometimes failed to sleep or lost sleep | | | | | 1. Yes 2. No | |
| Q309 | | | | There were moments when I felt life was so tough that I cried or wanted to cry | | | | | 1. Yes 2. No | |
| Q310 | | | | I felt run down (tired) | | | | | 1. Yes 2. No | |
| Q311 | | | | At times I felt like committing suicide | | | | | 1. Yes 2. No | |
| Q312 | | | | I was generally unhappy with things that I would be doing each day | | | | | 1. Yes 2. No | |
| Q313 | | | | My work was lagging behind | | | | | 1. Yes 2. No | |
| Q314 | | | | I felt I had problems in deciding what to do | | | | | 1. Yes 2. No | |

| **SECTION 4- MENTAL HEALTH AND WELL-BEING- SDQ**  **For each item, please mark the box for Not True, Somewhat True or Certainly True. It would help us if you answered all items as best you can even if you are not absolutely certain. Please give your answers based on how things have been for you over the last six month.** | | |
| --- | --- | --- |
| Q401 | I try to be nice to other people. I care about their feelings. | 1. Not true 2. Somewhat true 3. Certainly true |
| Q402 | I am restless, I cannot stay still for long | 1. Not true 2. Somewhat true 3. Certainly true |
| Q403 | I get a lot of headaches, stomach-aches or sickness | 1. Not true 2. Somewhat true 3. Certainly true |
| Q404 | I usually share with others (food, games, pens etc.) | 1. Not true 2. Somewhat true 3. Certainly true |
| Q405 | I get very angry and often lose my temper | 1. Not true 2. Somewhat true 3. Certainly true |
| Q406 | I am usually on my own. I generally play alone or keep to myself | 1. Not true 2. Somewhat true 3. Certainly true |
| Q407 | I usually do as I am told | 1. Not true 2. Somewhat true 3. Certainly true |
| Q408 | I worry a lot | 1. Not true 2. Somewhat true 3. Certainly true |
| Q409 | I am helpful if someone is hurt, upset or feeling ill | 1. Not true 2. Somewhat true 3. Certainly true |
| Q410 | I am constantly fidgeting or squirming | 1. Not true 2. Somewhat true 3. Certainly true |
| Q411 | I have one good friend or more | 1. Not true 2. Somewhat true 3. Certainly true |
| Q412 | I fight a lot. I can make other people do what I want. | 1. Not true 2. Somewhat true 3. Certainly true |
| Q413 | I am often unhappy, down-hearted or tearful | 1. Not true 2. Somewhat true 3. Certainly true |
| Q414 | Other people my age generally like me | 1. Not true 2. Somewhat true 3. Certainly true |
| Q415 | I am easily distracted, I find it difficult to concentrate | 1. Not true 2. Somewhat true 3. Certainly true |
| Q416 | I am nervous in new situations. I easily lose confidence. | 1. Not true 2. Somewhat true 3. Certainly true |
| Q417 | I am kind to younger children. | 1. Not true 2. Somewhat true 3. Certainly true |
| Q418 | I am often accused of lying or cheating | 1. Not true 2. Somewhat true 3. Certainly true |
| Q419 | Other children or young people pick on me or bully me | 1. Not true 2. Somewhat true 3. Certainly true |
| Q420 | I often volunteer to help others (parents, teachers, children) | 1. Not true 2. Somewhat true 3. Certainly true |
| Q421 | I think before I do things | 1. Not true 2. Somewhat true 3. Certainly true |
| Q422 | I take things that are not mine from home, school or elsewhere | 1. Not true 2. Somewhat true 3. Certainly true |
| Q423 | I get on better with adults than with people my own age | 1. Not true 2. Somewhat true 3. Certainly true |
| Q424 | I have many fears, I am easily scared | 1. Not true 2. Somewhat true 3. Certainly true |
| Q425 | I finish the work I’m doing. My attention is good | 1. Not true 2. Somewhat true 3. Certainly true |
