# Supplementary material for: Mobile Phone Access and Implications for Digital Health Interventions Among Adolescents and Young Adults in Zimbabwe: Cross-Sectional Survey
Source: JMIR Mhealth Uhealth. 2021 Jan 13;9(1):e21244. doi: 10.2196/21244 (PMC7840276; doi:10.2196/21244)
Supplement: Multimedia Appendix 2 [file mhealth_v9i1e21244_app2.docx]

|  | Male (n=161) |  | Female  (n=262) |  | Total  (n=423) |  |
| --- | --- | --- | --- | --- | --- | --- |
|  | **%** | **CI** | **%** | **CI** | **%** | **CI** |
| Number of phones owned or shared |  |  |  |  |  |  |
| 1 | 81.4 | [73.8,87.1] | 82.4 | [76.5,87.1] | 82 | [76.8,86.3] |
| 2 | 15.5 | [10.3,22.8] | 13.7 | [9.8,19.0] | 14.4 | [10.8,19.0] |
| 3+ | 3.1 | [1.4,6.9] | 3.8 | [1.8,7.9] | 3.5 | [2.0,6.1] |
| Total | 100 |  | 100 |  | 100 |  |
| Number of phone numbers currently used |  |  |  |  |  |  |
| 0 | 1.2 | [0.2,8.1] | 3.8 | [1.8,7.8] | 2.8 | [1.5,5.5] |
| 1 | 72.7 | [65.6,78.8] | 69.5 | [63.7,74.7] | 70.7 | [66.0,75.0] |
| 2 | 25.5 | [19.0,33.2] | 23.3 | [18.3,29.2] | 24.1 | [20.1,28.6] |
| 3 | 0.6 | [0.1,4.4] | 3.4 | [1.9,6.2] | 2.4 | [1.3,4.2] |
| Total | 100 |  | 100 |  | 100 |  |
| Number of phones numbers used past year (categorised) |  |  |  |  |  |  |
| 0 | 1.2 | [0.2,8.1] | 3.4 | [1.7,7.0] | 2.6 | [1.3,5.0] |
| 1 | 57.1 | [47.0,66.7] | 53.1 | [45.6,60.4] | 54.6 | [47.9,61.2] |
| 2 | 32.9 | [25.3,41.6] | 32.1 | [26.4,38.3] | 32.4 | [27.4,37.8] |
| 3+ | 8.7 | [5.0,14.8] | 11.5 | [7.7,16.6] | 10.4 | [7.6,14.0] |
| Is main owned phone a smart phone? (n=396) |  |  |  |  |  |  |
| No | 9.2 | [5.1,15.9] | 18.9 | [14.6,24.1] | 15.2 | [12.1,18.9] |
| Yes | 90.8 | [84.1,94.9] | 81.1 | [75.9,85.4] | 84.8 | [81.1,87.9] |
| Is main shared phone a smart phone? (n=70) |  |  |  |  |  |  |
| No | 25.0 | [7.3,58.5] | 12.0 | [5.8,23.2] | 15.7 | [7.7,29.5] |
| Yes | 75.0 | [41.5,92.7] | 88.0 | [76.8,94.2] | 84.3 | [70.5,92.3] |
| Year main owned phone purchased (n=396) |  |  |  |  |  |  |
| 2015 or earlier | 5.2 | [2.7,10.0] | 6.2 | [4.0,9.5] | 5.8 | [3.9,8.5] |
| 2016 | 7.8 | [4.5,13.4] | 14.4 | [10.4,19.6] | 11.9 | [9.2,15.2] |
| 2017 | 27.5 | [20.9,35.2] | 30.0 | [25.0,35.7] | 29.0 | [24.7,33.8] |
| 2018 | 56.2 | [47.4,64.7] | 46.1 | [40.2,52.1] | 50.0 | [44.8,55.2] |
| Not known | 3.3 | [1.4,7.5] | 3.3 | [1.7,6.4] | 3.3 | [1.9,5.7] |
| Who respondent shares main phone with (n=70) |  |  |  |  |  |  |
| Sister | 25.0 | [8.6,54.3] | 14.0 | [6.6,27.3] | 17.1 | [9.2,29.6] |
| Brother | 30.0 | [13.4,54.2] | 10.0 | [3.4,25.9] | 15.7 | [7.8,29.2] |
| Mother | 20.0 | [6.5,47.5] | 36.0 | [22.3,52.4] | 31.4 | [19.0,47.2] |
| Other adult in household | 5.0 | [0.6,30.4] | 2.0 | [0.2,15.0] | 2.9 | [0.7,11.5] |
| Friend | 20 | [6.5,47.4] | 6.0 | [2.1,16.1] | 10.0 | [4.6,20.4] |
| Partner/boyfriend/girlfriend | 0.0 |  | 28.0 | [15.8,44.7] | 20.0 | [11.5,32.5] |
| Other | 0.0 |  | 4.0 | [1.0,15.1] | 2.9 | [0.7,11.2] |
| Frequency of access to main shared phone (n=70) |  |  |  |  |  |  |
| at least once/day | 80.0 | [52.8,93.5] | 70.0 | [54.6,81.9] | 72.9 | [59.2,83.2] |
| at least once/week | 15.0 | [4.1,42.1] | 26.0 | [15.1,41.0] | 22.9 | [13.5,36.0] |
| Less than once a week | 5.0 | [0.6,31.5] | 4.0 | [0.9,16.8] | 4.3 | [1.3,13.6] |
