# Supplementary material for: Mobile Phone Access and Implications for Digital Health Interventions Among Adolescents and Young Adults in Zimbabwe: Cross-Sectional Survey
Source: JMIR Mhealth Uhealth. 2021 Jan 13;9(1):e21244. doi: 10.2196/21244 (PMC7840276; doi:10.2196/21244)
Supplement: Multimedia Appendix 3 [file mhealth_v9i1e21244_app3.docx]

|  | Male (n=75) |  | Female (n=135) |  | Total (n=210) |  |
| --- | --- | --- | --- | --- | --- | --- |
|  | **%** | **CI** | **%** | **CI** | **%** | **CI** |
| Why doesn’t have a mobile phone |  |  |  |  |  |  |
| It is not allowed | 18.7 | [10.4,31.3] | 16.3 | [10.0,25.4] | 17.1 | [11.8,24.3] |
| Doesn’t require it | 2.7 | [0.7,10.3] | 3.7 | [1.5,8.9] | 3.3 | [1.6, 7.0] |
| It is costly | 22.7 | [13.9,34.8] | 21.5 | [15.1,29.6] | 21.9 | [16.4,28.6] |
| Has/had a phone but it is not working | 33.3 | [24.0,44.2] | 24.4 | [18.1,32.1] | 27.6 | [22.1,34.0] |
| Other | 22.7 | [14.0,34.5] | 34.1 | [24.6, 45.0] | 30 | [23.0, 38.0] |
| Planning to buy a mobile phone in the near future? |  |  |  |  |  |  |
| No | 24.0 | [15.3, 35.6] | 28.9 | [22.5, 36.3] | 27.1 | [21.4, 33.8] |
| Yes | 66.7 | [55.9, 75.9] | 62.2 | [54.7, 69.3] | 63.8 | [57.6, 69.6] |
| Don’t know/no response | 9.3 | [3.8, 21.3] | 8.9 | [5.1, 15.1] | 9.0 | [5.8, 13.9] |
| When plan to buy phone |  |  |  |  |  |  |
| Within the next month (already in process) | 24.0 | [14.6, 36.9] | 25.0 | [16.4, 36.3] | 24.6 | [17.6, 33.3] |
| Sometime in the next 3-6 months | 22.0 | [12.5, 35.8] | 17.9 | [10.9, 27.9] | 19.4 | [13.3, 27.5] |
| Maybe when I start working/earning | 52.0 | [38.4, 65.4] | 47.6 | [34.7, 60.8] | 49.3 | [39.5, 59.0] |
| Maybe when I start going to secondary school | 2.0 | [0.3, 12.7] | 9.5 | [4.6, 18.5] | 6.7 | [3.2, 13.5] |
