# Supplementary material for: Mobile Phone Access and Implications for Digital Health Interventions Among Adolescents and Young Adults in Zimbabwe: Cross-Sectional Survey
Source: JMIR Mhealth Uhealth. 2021 Jan 13;9(1):e21244. doi: 10.2196/21244 (PMC7840276; doi:10.2196/21244)
Supplement: Multimedia Appendix 4 [file mhealth_v9i1e21244_app4.docx]

|  | Male (n=161) |  | Female (n=262) |  | Total (n=423) |  |
| --- | --- | --- | --- | --- | --- | --- |
|  | **%** | **CI** | **%** | **CI** | **%** | **CI** |
| Age group (years) when starting using mobile phone |  |  |  |  |  |  |
| <13y | 31.7 | [24.6,39.8] | 18.3 | [14.2,23.4] | 23.4 | [19.4,27.9] |
| 13-14y | 30.4 | [23.2,38.7] | 25.6 | [20.2,31.8] | 27.4 | [23.0,32.3] |
| 15-16y | 28.6 | [22.0,36.2] | 31.7 | [27.1,36.7] | 30.5 | [26.6,34.7] |
| 17+y | 8.7 | [5.5,13.5] | 22.9 | [18.2,28.4] | 17.5 | [14.3,21.2] |
| Don't know/no response | 0.6 | [0.1,4.2] | 1.5 | [0.6,4.0] | 1.2 | [0.5,2.8] |
| Why got first phone (n=367) |  |  |  |  |  |  |
| Everyone around you had one | 2.9 | [1.1,7.2] | 3.1 | [1.3,6.9] | 3 | [1.5,5.7] |
| You wanted one | 16.5 | [10.5,25.2] | 27.6 | [20.6,36.0] | 23.4 | [17.7,30.4] |
| Somebody (friend/parents) asked you to get one for yourself | 4.3 | [1.7,10.6] | 3.5 | [1.5,7.8] | 3.8 | [1.8,7.8] |
| Passed on to me | 37.4 | [28.1,47.8] | 23.7 | [17.6,31.0] | 28.9 | [23.0,35.6] |
| Gifted to me | 31.7 | [23.0,41.8] | 34.6 | [28.0,41.9] | 33.5 | [27.8,39.8] |
| Extra set available | 0 |  | 1.3 | [0.4,4.1] | 0.8 | [0.3,2.5] |
| Convenience | 5 | [1.9,12.7] | 1.8 | [0.7,4.5] | 3 | [1.5,6.0] |
| Work required it | 0.7 | [0.1,4.8] | 0.4 | [0.1,3.1] | 0.5 | [0.1,2.2] |
| Other | 1.4 | [0.3,5.8] | 3.9 | [1.8,8.5] | 3 | [1.4,6.2] |
| Who bought first phone (n=367) |  |  |  |  |  |  |
| Respondent | 8.6 | [4.9,14.7] | 8.3 | [5.2,13.0] | 8.4 | [5.7,12.4] |
| Parents | 64.7 | [56.8,72.0] | 64.5 | [57.6,70.8] | 64.6 | [59.6,69.2] |
| Other relative | 23.7 | [17.7,31.1] | 21.9 | [17.0,27.8] | 22.6 | [18.5,27.3] |
| Friend | 0.7 | [0.1,5.1] | 1.3 | [0.4,4.1] | 1.1 | [0.4,2.9] |
| Boyfriend/girlfriend | 0.7 | [0.1,5.1] | 2.6 | [1.2,5.7] | 1.9 | [0.9,3.9] |
| Other | 1.4 | [0.4,5.4] | 1.3 | [0.3,5.4] | 1.4 | [0.5,3.6] |
