# Supplementary material for: Mobile Phone Access and Implications for Digital Health Interventions Among Adolescents and Young Adults in Zimbabwe: Cross-Sectional Survey
Source: JMIR Mhealth Uhealth. 2021 Jan 13;9(1):e21244. doi: 10.2196/21244 (PMC7840276; doi:10.2196/21244)
Supplement: Multimedia Appendix 5 [file mhealth_v9i1e21244_app5.docx]

|  |  | male (n=139) | |  | female (n=228) | |  | Total (n=367) | |
| --- | --- | --- | --- | --- | --- | --- | --- | --- | --- |
|  | No. | % | CI | No. | % | CI | No. | % | CI |
| Phone ever switched off |  |  |  |  |  |  |  |  |  |
| Yes | 78 | 56.1 | [46.4,65.4] | 142 | 62.3 | [54.5,69.5] | 220 | 59.9 | [53.4,66.2] |
| No | 61 | 43.9 | [34.6,53.6] | 86 | 37.7 | [30.5,45.5] | 147 | 40.1 | [33.8,46.6] |
| Do you take your cellphone to school? (n=128) | |  |  |  |  |  |  |  |  |
| Yes, everyday | 13 | 21.7 | [13.2,33.5] | 17 | 25 | [16.3,36.3] | 30 | 23.4 | [17.2,31.1] |
| Yes, sometimes | 18 | 30 | [19.5,43.1] | 18 | 26.5 | [16.5,39.7] | 36 | 28.1 | [20.4,37.4] |
| No | 29 | 48.3 | [36.2,60.7] | 33 | 48.5 | [36.2,61.0] | 62 | 48.4 | [39.6,57.4] |
| Can you do without your cellphone for a day? | |  |  |  |  |  |  |  |  |
| No | 46 | 33.1 | [26.1,41.0] | 57 | 25 | [19.2,31.9] | 103 | 28.1 | [23.0,33.7] |
| Yes | 93 | 66.9 | [59.0,73.9] | 171 | 75 | [68.1,80.8] | 264 | 71.9 | [66.3,77.0] |
| Best thing about having a mobile phone |  |  |  |  |  |  |  |  |  |
| It is stylish | 3 | 2.2 | [0.7,6.6] | 8 | 3.5 | [1.6,7.4] | 11 | 3 | [1.6,5.6] |
| It makes your life easier/is convenient | 128 | 92.1 | [86.6,95.5] | 207 | 90.8 | [85.6,94.2] | 335 | 91.3 | [87.7,93.9] |
| You feel safer | 2 | 1.4 | [0.4,5.7] | 3 | 1.3 | [0.4,4.0] | 5 | 1.4 | [0.6,3.2] |
| Other | 6 | 4.3 | [2.0,9.0] | 10 | 4.4 | [2.5,7.7] | 16 | 4.4 | [2.7,6.9] |
