# Supplementary material for: Mobile Phone Access and Implications for Digital Health Interventions Among Adolescents and Young Adults in Zimbabwe: Cross-Sectional Survey
Source: JMIR Mhealth Uhealth. 2021 Jan 13;9(1):e21244. doi: 10.2196/21244 (PMC7840276; doi:10.2196/21244)
Supplement: Multimedia Appendix 6 [file mhealth_v9i1e21244_app6.docx]

|  | male (n=161) | | female (n=262) | | Total (n=423) | |
| --- | --- | --- | --- | --- | --- | --- |
|  | % | CI | % | CI | % | CI |
| Amount spent on airtime in past week -categorised |  |  |  |  |  |  |
| None | 10.6 | [6.2,17.4] | 9.5 | [6.2,14.3] | 9.9 | [7.1,13.7] |
| less than $4 | 65.2 | [56.8,72.8] | 66.8 | [60.5,72.5] | 66.2 | [60.8,71.2] |
| $4+ | 22.4 | [16.2,30.1] | 21 | [16.4,26.5] | 21.5 | [17.5,26.1] |
| Don't know/no response | 1.9 | [0.6,5.7] | 2.7 | [1.2,6.1] | 2.4 | [1.2,4.6] |
| Expenditure on airtime compared to other things in the past week |  |  |  |  |  |  |
| airtime less than other expenditure | 64.0 | [55.8,71.4] | 58.0 | [50.5,65.2] | 60.3 | [54.3,66.0] |
| airtime same as other expenditure | 14.3 | [9.8,20.4] | 16.8 | [12.6,22.0] | 15.8 | [12.5,19.9] |
| airtime more than other expenditure | 21.7 | [16.0,28.8] | 25.2 | [19.8,31.4] | 23.9 | [19.5,28.9] |
| Who usually pays for expenditure on mobile phone? ^b^ |  |  |  |  |  |  |
| Respondent only | 23.0 | [16.6,30.9] | 5.8 | [3.6,9.3] | 12.4 | [9.3,16.4] |
| Respondent and family member | 21.7 | [16.0,28.9] | 11.7 | [8.5,15.8] | 15.6 | [12.4,19.3] |
| Respondent and friend^a^ | 9.3 | [5.6,15.0] | 14 | [10.1,19.2] | 12.2 | [9.2,16.1] |
| Family member only | 17.4 | [12.4,23.9] | 16.7 | [12.5,22.1] | 17.0 | [13.5,21.2] |
| Friend only | 0 |  | 7.8 | [4.7,12.7] | 4.8 | [2.9,7.9] |
| Family and friend | 5.0 | [2.5,9.7] | 11.3 | [7.6,16.4] | 8.9 | [6.3,12.2] |
| Respondent, family, friend | 23.6 | [17.3,31.3] | 32.7 | [26.0,40.2] | 29.2 | [23.9,35.2] |
| Boyfriend/girlfriend usually pays for phone credit^2^ |  |  |  |  |  |  |
| No | 75.2 | [67.4, 81.6] | 47.1 | [41.6, 52.7] | 57.9 | [53.0, 62.7] |
| Yes | 24.8 | [18.4, 32.6] | 52.9 | [47.3, 58.5] | 42.1 | [37.3, 47.0] |

^a^Friend includes boyfriend or girlfriend, ^b^Missing for n=5 who own or share a phone
