# Supplementary material for: Mobile Phone Access and Implications for Digital Health Interventions Among Adolescents and Young Adults in Zimbabwe: Cross-Sectional Survey
Source: JMIR Mhealth Uhealth. 2021 Jan 13;9(1):e21244. doi: 10.2196/21244 (PMC7840276; doi:10.2196/21244)
Supplement: Multimedia Appendix 7 [file mhealth_v9i1e21244_app7.docx]

|  | Never/seldom | Sometimes | Often/always |
| --- | --- | --- | --- |
|  |  |  |  |
| Clock | 15.84 | 6.86 | 77.3 |
| Instant messages/chat | 16.55 | 8.51 | 74.94 |
| Camera | 23.4 | 18.91 | 57.68 |
| Calendar | 27.19 | 16.78 | 56.03 |
| Listen/download music | 30.73 | 16.31 | 52.96 |
| Bible | 42.55 | 18.68 | 38.77 |
| Play games | 50.83 | 15.84 | 33.33 |
| Watch TV/videos | 47.52 | 19.15 | 33.33 |
| Payments/purchases | 40.43 | 27.9 | 31.68 |
| Dictionary | 54.61 | 17.02 | 28.37 |
| Social networking | 54.61 | 18.91 | 26.48 |
| School/work | 57.68 | 16.08 | 26.24 |
| Send messages (not app) | 55.79 | 18.44 | 25.77 |
| Calculator | 45.39 | 30.73 | 23.88 |
| Phone calls | 43.97 | 33.33 | 22.7 |
| Get news/sports updates | 73.7 | 10.19 | 16.11 |
| Search info health | 74.47 | 16.31 | 9.22 |
| Search info relations | 79.67 | 12.06 | 8.27 |
| E-mailing | 86.49 | 9.24 | 4.27 |
| Navigate with maps | 89.81 | 7.35 | 2.84 |
