# Supplementary material for: Mobile Phone Access and Implications for Digital Health Interventions Among Adolescents and Young Adults in Zimbabwe: Cross-Sectional Survey
Source: JMIR Mhealth Uhealth. 2021 Jan 13;9(1):e21244. doi: 10.2196/21244 (PMC7840276; doi:10.2196/21244)
Supplement: Multimedia Appendix 8 [file mhealth_v9i1e21244_app8.docx]

|  | Never | Less than once/week | At least once/week |
| --- | --- | --- | --- |
| On a mobile phone | 8.1 | 13.7 | 78.2 |
| On a computer at school/work | 69.0 | 12.4 | 18.6 |
| on computer at commercial internet outlet | 84.2 | 10.3 | 5.6 |
| On a computer at home | 86.5 | 6.6 | 6.8 |
| on computer at someone else's house | 87.4 | 8.3 | 4.3 |
| on a computer in library/ community facility | 91.9 | 4.1 | 4.1 |
