# Supplementary material for: Mobile Phone Access and Implications for Digital Health Interventions Among Adolescents and Young Adults in Zimbabwe: Cross-Sectional Survey
Source: JMIR Mhealth Uhealth. 2021 Jan 13;9(1):e21244. doi: 10.2196/21244 (PMC7840276; doi:10.2196/21244)
Supplement: Multimedia Appendix 9 [file mhealth_v9i1e21244_app9.docx]

|  | Have at home | | | Ever used | | |
| --- | --- | --- | --- | --- | --- | --- |
|  | No. | % | CI | No. | % | CI |
| Desktop computer | 97 | 15.3 | [11.9, 19.5] | 391 | 61.7 | [56.5, 66.6] |
| Laptop | 173 | 27.3 | [22.8, 32.3] | 416 | 65.6 | [61.4, 69.6] |
| Tablet/ipad | 114 | 18.0 | [14.2, 22.5] | 306 | 48.3 | [44.0, 52.6] |
| Feature (non-smart) phone | 73 | 11.5 | [8.7, 15.1] | 168 | 26.5 | [21.1, 32.7] |
| Basic (call and SMS only) phone | 395 | 62.3 | [55.3, 68.8] | 515 | 81.2 | [76.6, 85.2] |
| Smart phone | 498 | 78.6 | [73.1, 83.2] | 538 | 84.9 | [81.4, 87.8] |
| Any mobile phone | 555 | 87.5 | [82.3, 91.4] | 591 | 93.2 | [90.3, 95.3] |
| ipod/mp3 player | 110 | 17.4 | [13.7, 21.8] | 259 | 40.9 | [36.5, 45.4] |
| TV | 565 | 89.1 | [86.1, 91.6] | 614 | 96.9 | [94.9, 98.1] |
| Radio | 451 | 71.1 | [66.5, 75.4] | 569 | 89.8 | [86.5, 92.3] |
| Digital camera | 58 | 9.1 | [6.9, 12.0] | 135 | 21.3 | [17.9, 25.1] |
| Gaming console | 60 | 9.5 | [7.0, 12.6] | 168 | 26.5 | [22.7, 30.7] |
| Handheld gaming device | 33 | 5.2 | [3.5, 7.7] | 111 | 17.5 | [14.1, 21.6] |
