# Supplementary material for: Mobile Phone Access and Implications for Digital Health Interventions Among Adolescents and Young Adults in Zimbabwe: Cross-Sectional Survey
Source: JMIR Mhealth Uhealth. 2021 Jan 13;9(1):e21244. doi: 10.2196/21244 (PMC7840276; doi:10.2196/21244)
Supplement: Multimedia Appendix 10 [file mhealth_v9i1e21244_app10.docx]

|  |  | male (n=191) | |  | female (n=274) | |  | Total (n=465) | |
| --- | --- | --- | --- | --- | --- | --- | --- | --- | --- |
|  | **No.** | **%** | **CI** | **No.** | **%** | **CI** | **No.** | **%** | **CI** |
| Most like about the internet |  |  |  |  |  |  |  |  |  |
| Entertainment | 65 | 34.0 | [27.3, 41.5] | 56 | 20.4 | [15.6, 26.3] | 121 | 26.0 | [21.6, 31.0] |
| Communication with lots of people e.g. | 33 | 17.3 | [12.1, 24.1] | 64 | 23.4 | [18.3, 29.4] | 97 | 20.9 | [16.8, 25.7] |
| 1:1 communication with friends/relatives | 22 | 11.5 | [7.7, 16.9] | 59 | 21.5 | [15.8, 28.6] | 81 | 17.4 | [13.3, 22.5] |
| Easily find information | 30 | 15.7 | [11.0,22.0] | 57 | 20.8 | [15.6, 27.2] | 87 | 18.7 | [14.6, 23.7] |
| Education | 17 | 8.9 | [5.5, 14.1] | 16 | 5.8 | [3.3, 10.1] | 33 | 7.1 | [4.8, 10.4] |
| Helps with studies | 13 | 6.8 | [3.6, 12.5] | 13 | 4.7 | [2.5, 8.7] | 26 | 5.6 | [3.4, 9.0] |
| Job search/career advice | 4 | 2.1 | [0.8, 5.3] | 2 | 0.7 | [0.2, 2.9] | 6 | 1.3 | [0.5, 3.1] |
| Supporting business | 2 | 1.0 | [0.3, 4.1] | 4 | 1.5 | [0.6, 3.7] | 6 | 1.3 | [0.6, 2.7] |
| Shopping | 0 | 0.0 | [0,0] | 1 | 0.4 | [0.1, 2.6] | 1 | 0.2 | [0.0, 1.5] |
| Other | 5 | 2.6 | [1.0, 7.0] | 2 | 0.7 | [0.2, 2.9] | 7 | 1.5 | [0.7, 3.4] |
| Most dislike about the internet |  |  |  |  |  |  |  |  |  |
| Nothing | 59 | 30.9 | [24.4, 38.2] | 103 | 37.6 | [31.7, 43.9] | 162 | 34.8 | [30.9, 39.9] |
| Unwanted sexual content seen | 56 | 29.3 | [23.3, 36.1] | 70 | 25.6 | [20.5, 31.3] | 126 | 27.1 | [22.9, 31.8] |
| Violent stories, photos, videos | 32 | 16.8 | [11.8, 23.3] | 29 | 10.6 | [7.0, 15.7] | 61 | 13.1 | [9.6, 17.6] |
| Too many adverts | 19 | 9.9 | [6.0, 16.1] | 33 | 12.0 | [8.1, 17.5] | 52 | 11.2 | [8.0, 15.4] |
| Scams | 9 | 4.7 | [2.5, 8.6] | 8 | 2.9 | [1.5, 5.6] | 17 | 3.6 | [2.3, 5.7] |
| Bullying | 0 | 0.0 | [0,0] | 5 | 1.8 | [0.8, 4.3] | 5 | 1.1 | [0.4, 2.6] |
| People share embarrassing things about me | 2 | 1.0 | [0.3, 4.0] | 3 | 1.1 | [0.4, 3.3] | 5 | 1.1 | [0.5, 2.5] |
| Other | 14 | 7.3 | [4.5, 11.7] | 23 | 8.4 | [5.4, 12.8] | 37 | 8.0 | [5.8, 10.8] |
| One thing that you would change to make the internet better |  |  |  |  |  |  |  |  |  |
| Cheaper data plans | 65 | 34.0 | [27.1, 41.7] | 106 | 38.7 | [31.8, 46.0] | 171 | 36.8 | [31.3, 42.7] |
| No change needed | 21 | 11.0 | [6.6, 17.7] | 64 | 23.4 | [17.3, 30.7] | 85 | 18.3 | [13.7, 24.0] |
| Easier access to mobile phones and computers | 32 | 16.8 | [11.8, 23.3] | 36 | 13.1 | [9.5, 18.0] | 68 | 14.6 | [11.4, 18.6] |
| Better internet coverage | 26 | 13.6 | [8.9, 20.2] | 34 | 12.4 | [8.1, 18.5] | 60 | 12.9 | [9.0, 18.1] |
| Higher speed connectivity | 33 | 17.3 | [12.4, 23.5] | 26 | 9.5 | [6.3, 14.1] | 59 | 12.7 | [9.9, 16.1] |
| Other | 14 | 7.3 | [3.9, 13.4] | 8 | 2.9 | [1.4, 6.0] | 22 | 4.7 | [2.7, 8.1] |

**^a^** Excludes Community E where 3/10 participants had ever used the internet
